# Silibinin sensitizes TRAIL-mediated apoptosis by upregulating DR5 through ROS-induced endoplasmic reticulum stress-Ca^2+^-CaMKII-Sp1 pathway

**DOI:** 10.18632/oncotarget.23129

**Published:** 2017-12-07

**Authors:** Matharage Gayani Dilshara, Rajapaksha Gedara Prasad Tharanga Jayasooriya, Ilandarage Menu Neelaka Molagoda, Jin-Woo Jeong, Seungheon Lee, Sang Rul Park, Gi-Young Kim, Yung Hyun Choi

**Affiliations:** ^1^ Department of Marine Life Sciences, Jeju National University, Jeju 63243, Republic of Korea; ^2^ Department of Biochemistry, College of Oriental Medicine, Dong-Eui University, Busan 47227, Republic of Korea

**Keywords:** silibinin, TRAIL, DR5, ROS, ER stress

## Abstract

In this study, we addressed how silibinin enhances tumor necrosis factor-related apoptosis-inducing ligand (TRAIL)-mediated apoptosis in various cancer cells. Combined treatment with silibinin and TRAIL (silibinin/TRAIL) induced apoptosis accompanied by the activation of caspase-3, caspase-8, caspase-9, and Bax, and cytosolic accumulation of cytochrome c. Anti-apoptotic proteins such as Bcl-2, IAP-1, and IAP-2 were inhibited as well. Silibinin also triggered TRAIL-induced apoptosis in A549 cells through upregulation of death receptor 5 (DR5). Pretreatment with DR5/Fc chimeric protein and DR5-targeted small interfering RNA (siRNA) significantly blocked silibinin/TRAIL-mediated apoptosis in A549 cells. Furthermore, silibinin increased the production of reactive oxygen species (ROS), which led to the induction of TRAIL-mediated apoptosis through DR5 upregulation. Antioxidants such as *N*-acetyl-L-cysteine and glutathione reversed the apoptosis-inducing effects of TRAIL. Silibinin further induced endoplasmic reticulum (ER) stress as was indicated by the increase in ER marker proteins such as PERK, eIF2α, and ATF-4, which stimulate the expression of CCAAT/enhancer binding protein homologous protein (CHOP). CHOP-targeted siRNA eliminated the induction of DR5 and resulted in a significant decrease in silibinin/TRAIL-mediated apoptosis. We also found that silibinin/TRAIL-induced apoptosis was accompanied with intracellular influx of Ca^2+^, which was stimulated by ER stress and the Ca^2+^ chelator, ethylene glycol tetraacetic acid (EGTA). Ca^2+^/calmodulin-dependent protein kinase (CaMKII) inhibitor, K252a, blocked silibinin/TRAIL-induced DR5 expression along with TRAIL-mediated apoptosis. Accordingly, we showed that ROS/ER stress-induced CaMKII activated Sp1, which is an important transcription factor for DR5 expression. Our results showed that silibinin enhanced TRAIL-induced apoptosis by upregulating DR5 expression through the ROS-ER stress-CaMKII-Sp1 axis.

## INTRODUCTION

Tumor necrosis factor (TNF)-related apoptosis-inducing ligand (TRAIL) is considered as a potential therapeutic agent for cancer. This is because it is able to induce apoptosis of various cancer cells without cytotoxicity in the normal cells [1]. TRAIL is well-known to induce apoptosis via direct binding with death receptors (DR) 4 and/or 5, leading to the formation of the death-inducing signal complex by binding to caspase-8 [2, 3]. TRAIL-mediated apoptosis is suppressed by competitors of DR4 and/or DR5, known as decoy receptors (DcR1 and DcR2) [4]. TRAIL-induced apoptosis initiated by the extrinsic pathway involves the proteolytic activation of caspase-8 and subsequently, the activation of effector caspases including caspases-3 [5]. Activation of caspase-8 also converts Bid to truncated Bid (tBid), which activates Bax. This leads to dissipation of mitochondrial transmembrane potential and release of cytochrome *c*. In turn, caspase-9 is activated by mitochondria during the intrinsic apoptosis pathways [6]. Therefore, tBid, activated by caspase-8, translocates to the mitochondria and activates Bax, which provides the link between the extrinsic and the intrinsic apoptotic pathways [6]. Nevertheless, Zhang *et al*., reported that treatment with TRAIL alone may not be sufficient for killing certain types of malignant cells including lung cancer [7]. Therefore, recent studies have attempted to research novel anticancer drugs that can enhance the sensitivity (effective sensitizers) of cancer cells to TRAIL [8, 9], in order to overcome TRAIL resistance in cancer cells.

Reactive oxygen species (ROS) is a key stimulator in cell death by triggering DNA damage, resulting from caspase activation [10]. ROS is a foremost upstream signal molecule linked to the modulation of TRAIL signaling, which leads to increasing TRAIL sensitivity in various cancer cells by inducing the expression of DRs [10]. Watanabe *et al*. reported that oxidants activate intracellular signaling pathways through mitogen-activated kinases (MAPKs) in different cell types by stimulating ROS generation [11]. Recently, Sung *et al*., showed that gossypol enhances DR5 expression in colon cancer cells by stimulating the ROS-MAPKs-CCAAT/enhancer binding protein homologous protein (CHOP) pathway, leading to TRAIL-induced apoptosis [12]. Moreover, the endoplasmic reticulum (ER) is implicated in DR5 expression, which accordingly promotes TRAIL-induced apoptosis [13]. In our previous study, we showed in detail that protein kinase RNA-like ER kinase (PERK)-induced eIF2α phosphorylation upregulated CHOP-mediated DR5 expression, leading to increased sensitivity to TRAIL. This suggests that ER stress also upregulates DR5 expression and consequently promotes TRAIL-induced apoptosis [14]. Prolonged or severe stress response under oxidative stress disturbs ER response, leading to increase in intracellular Ca^2+^ release, which can stimulate DR5 expression [15, 16]. However, ROS is not an outcome of the ER stress response [7], which suggests that crosstalk between ROS and ER-mediated Ca^2+^ release, stimulates DR5 expression. This sensitizes TRAIL-induced apoptosis by binding with DR5.

Silibinin is a major bioactive component of silymarin, and possesses chemopreventive properties against various cancers such as those of the colon, prostate, breast, as well gliomas [17, 18]. Silibinin-induced cell death depends on ROS-induced caspase-3 activation, resulting from inhibition of Notch-1 signaling and consequent inhibition of the MAPK pathway [19]. Silibinin also induces autophagic cell death via ROS-dependent deregulation of mitochondrial activities and depletion in ATP [17]. These studies indicate that silibinin is a potent candidate, which induces apoptosis of cancer cells by promoting ROS generation. Nevertheless, Kosteck *et al*. revealed that silibinin protected hepatocytes from various toxins, resulting from a reduction in ROS generation via GSH accumulation and peroxidase activation, and stimulated regeneration of the liver [20]. Although the reason for this discrepancy is not clear, the multifunctional effect of silibinin extends to cancers, diabetes, and neuroprotection.

In the present study, we investigated the potential of silibinin to enhance TRAIL-mediated apoptosis in human lung cancer A549 cells by promoting DR5 expression. ROS-induced ER response is a major hallmark in silibinin/TRAIL-induced apoptosis. The response stimulates Ca^2+^-activated CaMKII, leading to upregulation of DR5 through direct and/or indirect activation of Sp1. From our results, silibinin is a promising candidate as a chemotherapeutic agent, since it overcomes the resistance of cancer cells to TRAIL sensitivity.

## RESULTS

### Silibinin enhances TRAIL-induced apoptosis in various cancer cells

**Figure 1:**
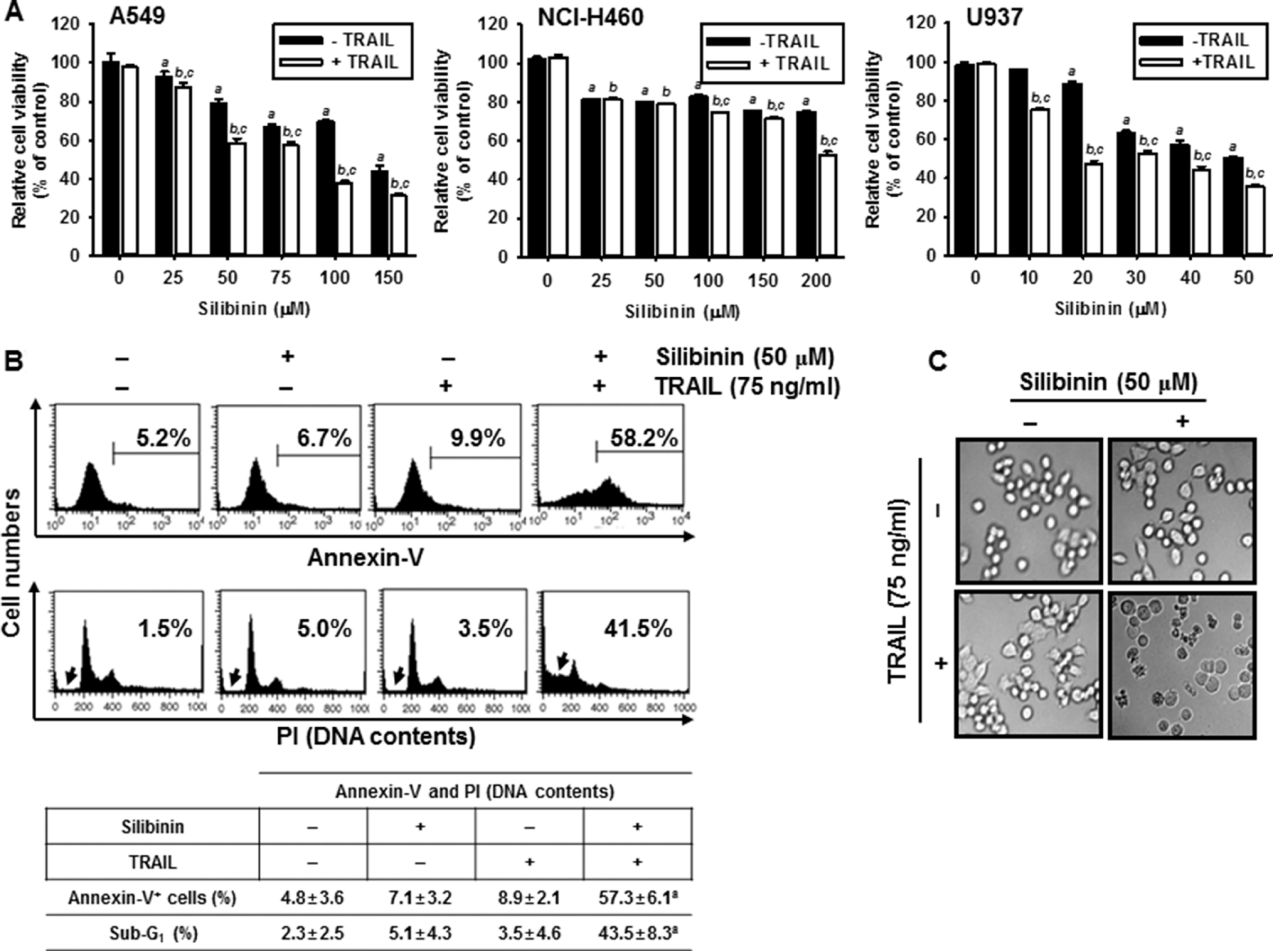
Effect of silibinin and/or TRAIL on cancer cell viability Human lung cancer (A549 and NCI-H460) cell lines and human leukemia U937 cells were treated with the indicated concentrations of silibinin for 1 h and then treated with or without TRAIL (75 ng/ml) for 24 h. (**A**) Cellular viability was determined by MTT assay. (**B**) In a parallel experiment, annexin-V^+^ population (top panel) and sub-G_1_ cell distribution (bottom panel) of A549 cells were analyzed by flow cytometry. (**C**) Cellular morphology with or without silibinin/TRAIL for 24 h was examined under light microscopy (400×). Data are representative of three independent experiments. Each point represents the mean ± S.E. of three independent experiments. Statistical significance was determined by a two-way ANOVA test (^*a*^ and ^b^, *p* < 0.05 *vs*. each untreated group and ^*c*^*p* < 0.05 *vs*. each respective group).

In order to investigate the effects of silibinin on the sensitivity of cancer cells to TRAIL, cell viability was analyzed in human lung cancer cell line A549 and NCI-H460, and human leukemia U937 cells. The cells were pretreated with silibinin for 1 h prior to treatment with TRAIL for 24 h. Cell viability was then determined by an MTT assay. TRAIL alone had no influence on cell viability in all the cell types; however, silibinin alone decreased cell viability in a dose-dependent manner (Figure 1A). The cells were sensitized to TRAIL (75 ng/ml) in the presence of silibinin. The percentage of viable cells at highest concentration of silibinin (200 μM) in the presence of TRAIL was less than 60% in all cell lines (Figure 1A). In particular, A549 and U937 cells were more sensitive to the combined treatment with silibinin and TRAIL (silibinin/TRAIL) than NCI-H460 cells. The results indicate that silibinin/TRAIL effectively inhibits cell viability in various cancer cells. We further investigated whether silibinin/TRAIL enhances apoptosis in A549 cells. Silibinin (50 μM)/TRAIL (75 ng/ml) treatment for 24 h significantly increased annexin-V^+^ populations (top) and of sub-G_1_ phase cells (bottom) by approximately 58% of and 41%, respectively, whereas silibinin alone or TRAIL alone slightly changed the apoptotic population (Figure 1B). Silibinin/TRAIL also showed apoptotic bodies with a low number of cells compared to the untreated cells under phase contrast microscopy (Figure 1C).

**Figure 2:**
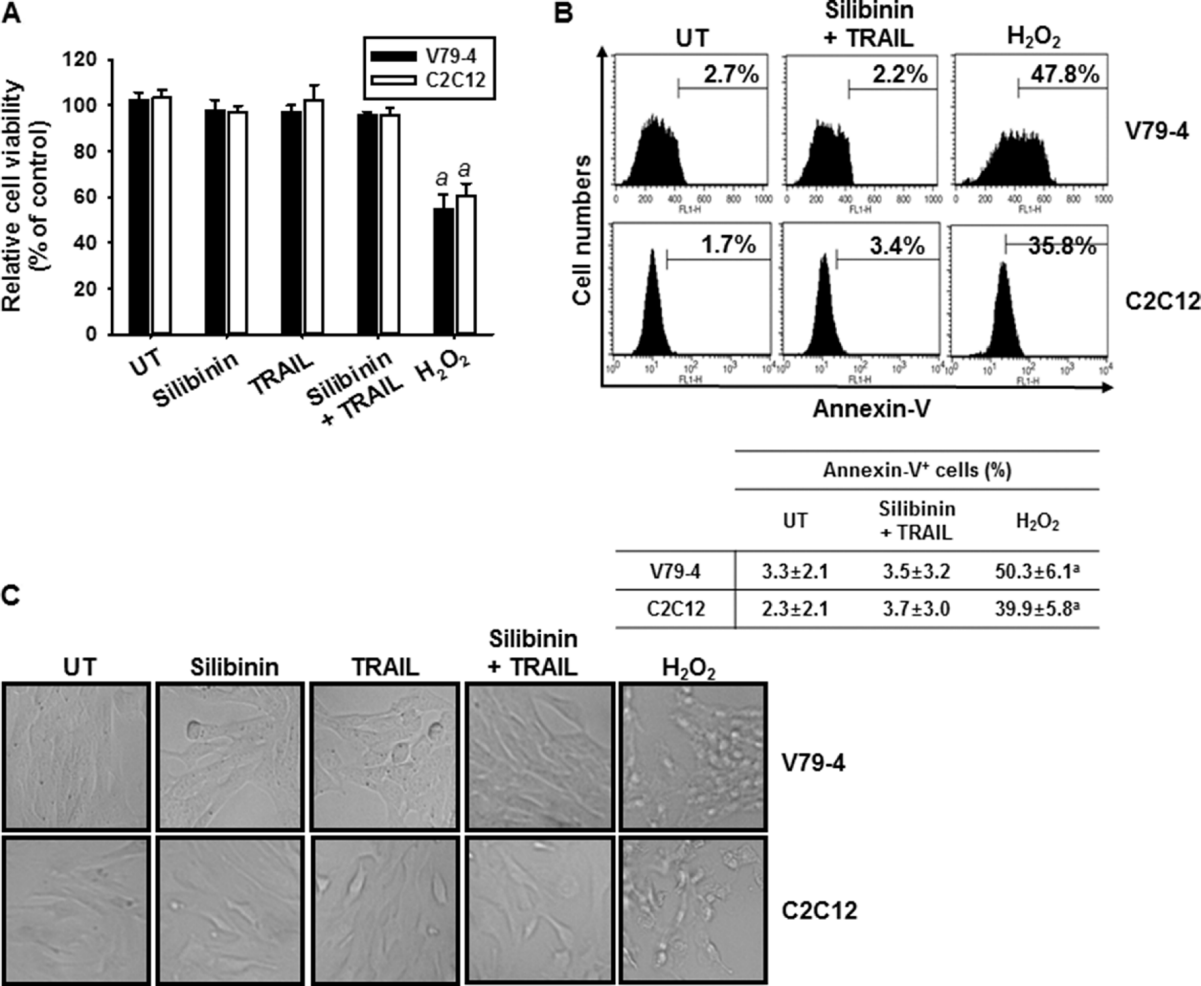
Effect of silibinin and TRAIL on cell viability of normal V79-4 and C2C12 cells Fibroblast V79-4 and myoblast C2C12 cells were treated with 50 μM silibinin for 1 h and then treated with or without TRAIL (75 ng/ml) for 24 h. (**A**) Cellular viability was determined by MTT assay. (**B**) In a parallel experiment, annexin-V^+^ population of V74-9 cells (top panel) and C2C12 cells (bottom panel) was analyzed by flow cytometry. (**C**) Cellular morphology with or without silibinin/TRAIL for 24 h was examined under light microscopy (400×). Data are representative of three independent experiments. Each point represents the mean ± S.E. of three independent experiments. Statistical significance was determined by a one-way ANOVA test (^*a*^*p* < 0.05 *vs*. each untreated group).

We also analyzed whether silibinin/TRAIL induces apoptosis in normal cell lines, fibroblast V79-4 and myoblast C2C12. As shown in Figure 2A, no relative cell viability was changed in the presence of silibinin/TRAIL and silibinin or TRAIL alone did not influence cell viability, compared to apoptosis-inducing positive control, H_2_O_2_ treatment. In addition, annexin-V staining (Figure 2B) and cell morphology (Figure 2C) confirmed that silibinin/TRAIL did not change cell viability in normal cell lines. Collectively, these data suggest that silibinin is a promising TRAIL sensitizer to overcome cell resistance to TRAIL.

### Silibinin/TRAIL induces apoptosis via extrinsic and intrinsic pathways

**Figure 3:**
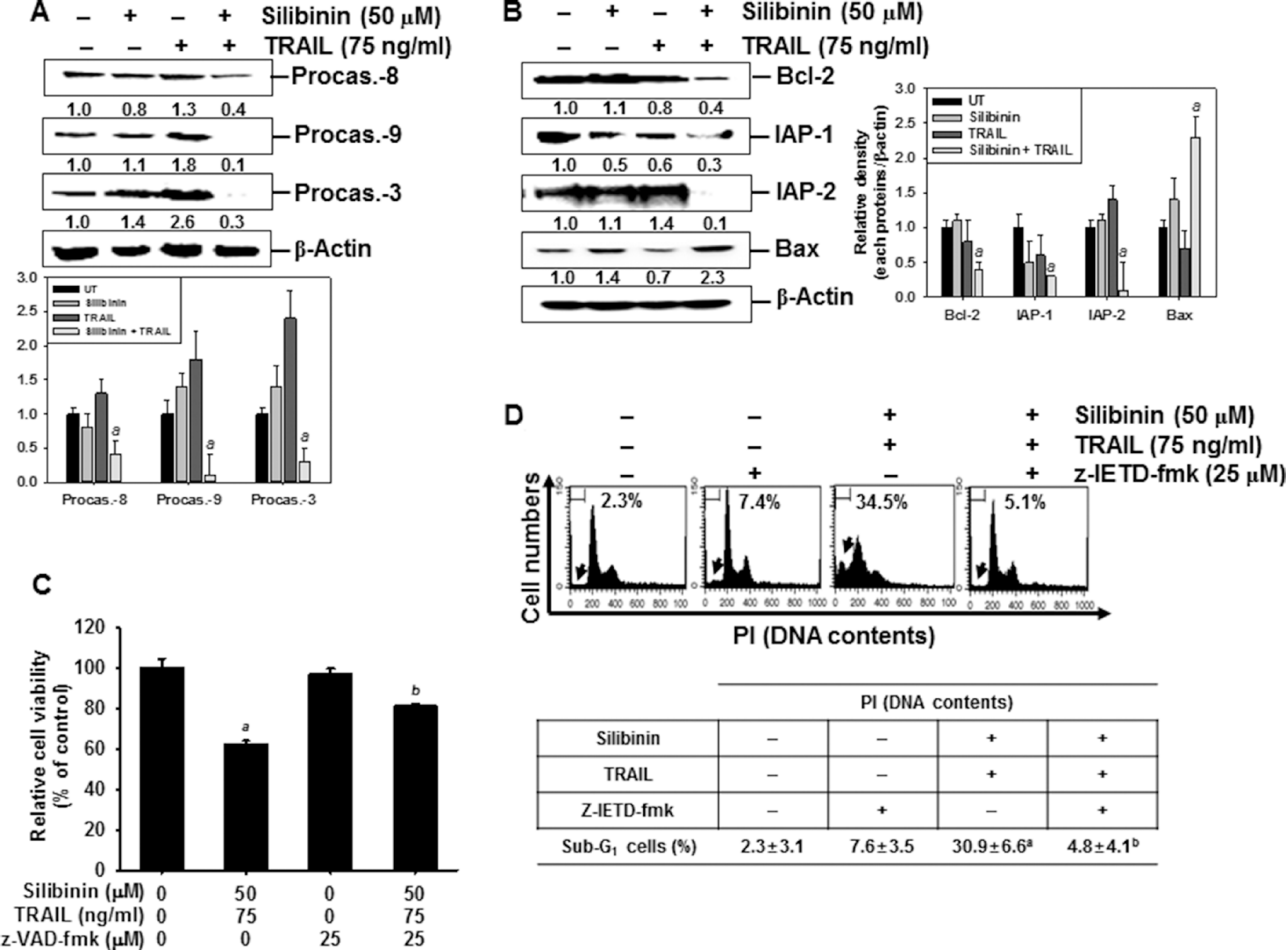
Effect of silibinin and/or TRAIL on the expression of intrinsic and extrinsic apoptotic pathway Human A549 cells were treated with 50 μM silibinin for 1 h and then treated with or without TRAIL (75 ng/ml) for 24 h. (**A**–**B**) Western blot analysis was performed for the expression of caspases (A) and apoptosis-regulating proteins such as Bcl-2, IAP-1, IAP-2, and Bax (B). (**C**) To clarify the effect of the inhibition of caspases, the cells were pretreated with 25 μM z-VAD-fmk for 30 min before combined treatment with silibinin/TRAIL for 24 h. Cellular viability was determined by MTT assay. (**D**) In a parallel experiment, A549 cells were pretreated with 25 μM z-IETD-fmk for 30 min and then silibinin and TRAIL were administrated. After 24 h-incubation, sub-G_1_ cell distribution was analyzed by flow cytometry. (**E**) Western blot analysis was performed for the expression of cytochrome *c* in the cytosol. (**F**) Mitochondrial membrane potential was analyzed by DiOC_6_ staining by flow cytometry. (**G**) The cellular morphology of cells with or without silibinin/TRAIL for 24 h was examined under light microscopy (400×). β-Actin was used as an internal control. Data are representative of three independent experiments. Each point represents the mean ± S.E. of three independent experiments. Statistical significance was determined by a two-way ANOVA test (^*a*^*p* < 0.05 *vs*. untreated group and ^*c*^*p* < 0.05 *vs*. silibinin/TRAIL-treated group).

To gain insight into the mechanism by which the apoptotic process is induced by silibinin/TRAIL, we performed western blot analyses for several caspases. Silibinin/TRAIL caused a significant decrease in the activations of procaspase-3, procaspase-8, and procaspase-9. However, the levels of the procaspases were not changed in response to either TRAIL alone or silibinin alone (Figure 3A). Additionally, silibinin/TRAIL significantly decreased the expressions of antiapoptotic Bcl-2, IAP-1, and IAP-2, and induced the apoptotic Bax (Figure 3B). Silibinin alone or TRAIL alone slightly changed all the proteins studied in this experiment.

Next, we checked the percentages of viable cells after silibinin/TRAIL-mediated apoptosis in the presence of the pan-caspase inhibitor, z-VAD-fmk. The presence of silibinin/TRAIL decreased cell viability (approximately 60%); however, pretreatment of the cells with z-VAD-fmk restored the silibinin/TRAIL-induced cell death by approximately 80% (Figure 3C). To verify the role of caspase-8 activation in silibinin/TRAIL-induced apoptosis, we used z-IETD-fmk, which is a caspase-8 inhibitor. Pretreating the cells with z-IETD-fmk completely inhibited an increase in silibinin/TRAIL-induced apoptosis. This suggested that caspase-8 induced apoptosis by activating the silibinin/TRAIL-mediated extrinsic signaling pathways (Figure 3D). Additionally, silibinin/TRAIL significantly facilitated the release of cytochrome c into cytosol, which then activated caspase-9 (Figure 3E). To verify the role of mitochondria in apoptosis by silibinin/TRAIL, mitochondrial membrane potential was determined using DiOC_6_. The mitochondrial membrane potential was significantly downregulated in response to silibinin/TRAIL. However, the presence of z-IETD-fmk restored silibinin/TRAIL-mediated mitochondrial membrane potential treatment to the control levels. This indicates that silibinin/TRAIL stimulates caspase-8-induced extrinsic pathway, leading to activation of caspase-9 via the intrinsic pathway (Figure 3F). The membrane potential was also slightly reduced due to treatment with silibinin alone or TRAIL alone. Additionally, silibinin/TRAIL resulted in the formation of apoptotic bodies and cell shrinkage. However, the presence of z-IETD-fmk blocked the morphological changes and the cells appeared as having a normal morphology (Figure 3G). These data imply that silibinin/TRAIL-induced apoptosis enhances crosstalk between the intrinsic and extrinsic pathways.

### Silibinin triggers DR5 upregulation, resulting in enhancement of TRAIL-mediated apoptosis

**Figure 4:**
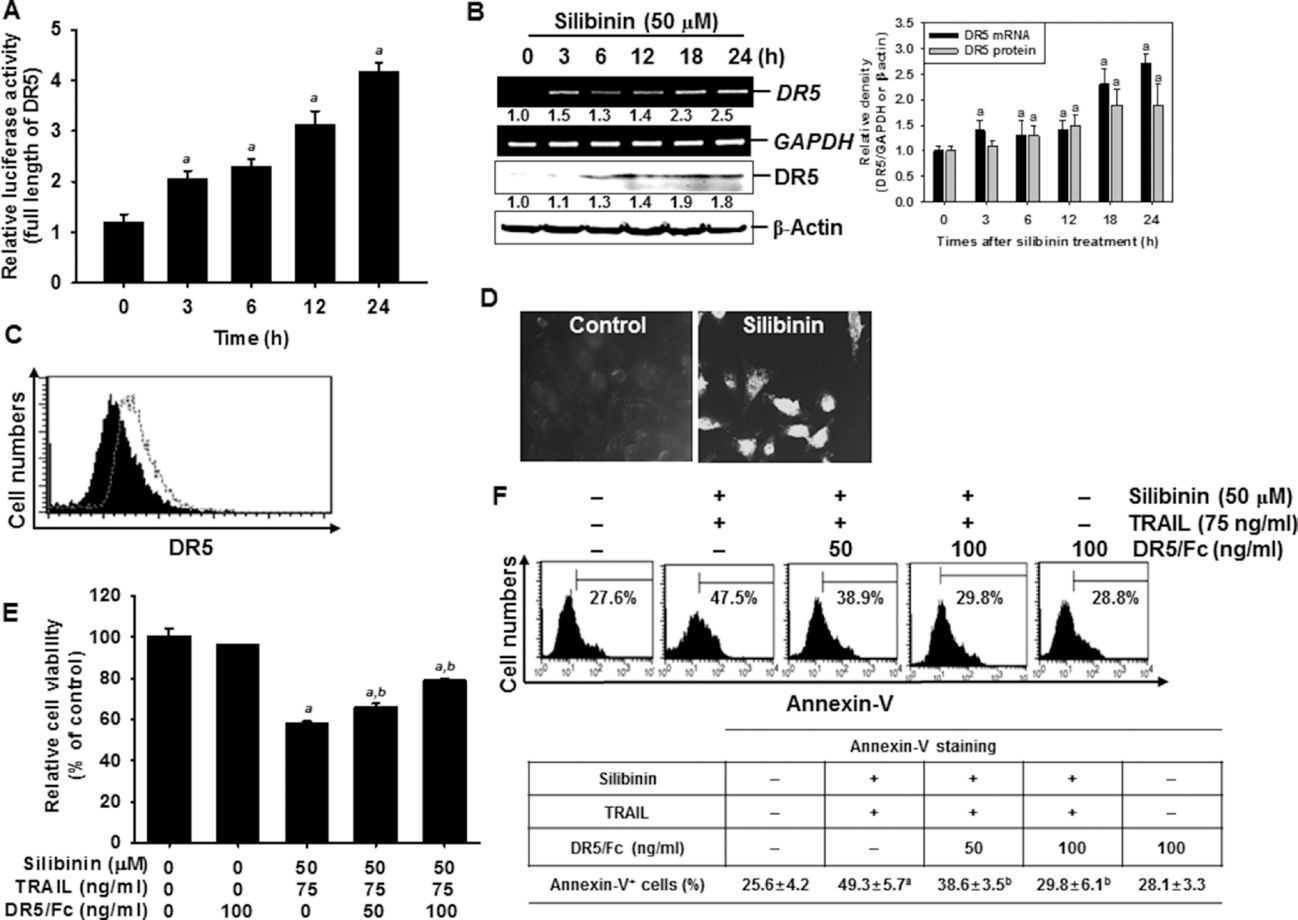
Sensitization of TRAIL-induced apoptosis by silibinin through DR5 upregulation A549 cells were treated with 50 μM silibinin for the incdicated time points. (**A**) The cells were transfected with the full length reporter constructs and lysates were assayed for luciferase activity of DR5 promoter. (**B**) Total RNA and total protein were isolated, and RT-PCR and wetern blot analysis were performed for DR5 expression. GAPDH and β-Actin were used as internal controls for RT-PCR and western blot analysis respectively. (**C**) The cell surface expression of DR5 in A549 cells was analyzed after silibinin treatment for 24 h by flow cytometry, after the cells were stained with goat anti-DR5 antibody. Cells were further incubated with FITC-conjugated rabbit anti-goat IgG. Black shade and grey dot show the untreated control and silibinin-treated group, respectively. (**D**) A549 cells were stained with goat anti-DR5 antibody and FITC-conjugated rabbit anti-goat IgG and analyzed by laser scanning microscopy. (**E**) A549 cells were treated with 50 μM silibinin and/or 75 ng/ml TRAIL in the presence of the indicated concentrations of the DR5 chimera proteins. After incubation for 24 h, cell viability was analyzed by MTT assay. (**F**) Annexin-V^+^ population of A549 cells were analyzed by flow cytometry. Each point represents the mean ± S.E. of three independent experiments. Statistical significance was determined by a two-way ANOVA test (^*a*^*p* < 0.05 *vs*. untreated group and ^*b*^*p* < 0.05 *vs*. silibinin/TRAIL-treated group).

The surface expression of DR5 is essential in the transmission of death signals to cells from the TRAIL ligand [2, 3]. Therefore, to elucidate the molecular mechanism underlying the silibinin-induced expression of DR5, reporter constructs containing full-lengths of DR5 gene promoter regions were examined in A549 cells. The results indicated that silibinin significantly increased the promoter activity of DR5 in a time-dependent manner (Figure 4A). At 24 h, the increase in DR5 promoter activity by silibinin was 4-fold higher than that by the control. This confirmed that silibinin-induced DR5 upregulation is controlled at the transcription level. Silibinin also gradually increased the mRNA (top) and protein (bottom) levels of DR5 in a time-dependent manner (Figure 4B). Consistent with the data from RT-PCR and western blot analysis, FACS analysis also revealed that silibinin increased the expression of DR5 on the surface of the cells (Figure 4C). Immunofluorescence also showed that there was a higher increment in the expression of DR5 on silibinin-treated cells, compared to that on the untreated control cells (Figure 4D). We used DR5/Fc chimeric protein to confirm the functional role of DR5 in silibinin/TRAIL-induced apoptosis. The data from the MTT assays showed that pretreating cells with DR5/Fc chimeric protein significantly inhibited silibinin/TRAIL-induced cell death (Figure 4E). According to the FACS analysis, silibinin/TRAIL increased the percentage of apoptotic cells whereas pretreatment with DR5/Fc chimeric protein markedly decreased silibinin/TRAIL-mediated apoptosis (Figure 4F). The results indicated that silibinin-induced upregulation of DR5 is required for the enhancement of TRAIL sensitivity.

### Silibinin potentiates TRAIL-mediated apoptosis through ROS generation

**Figure 5:**
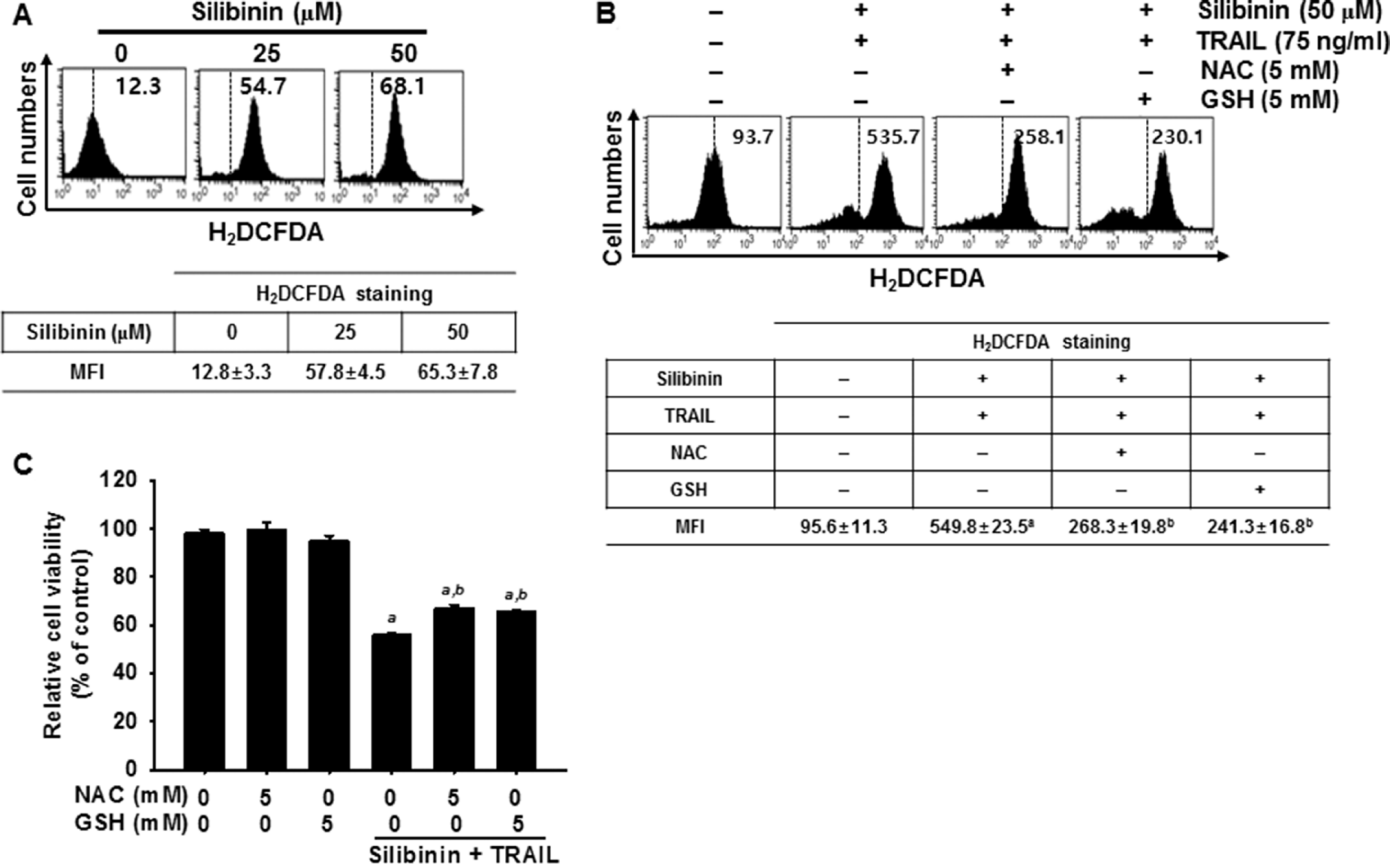

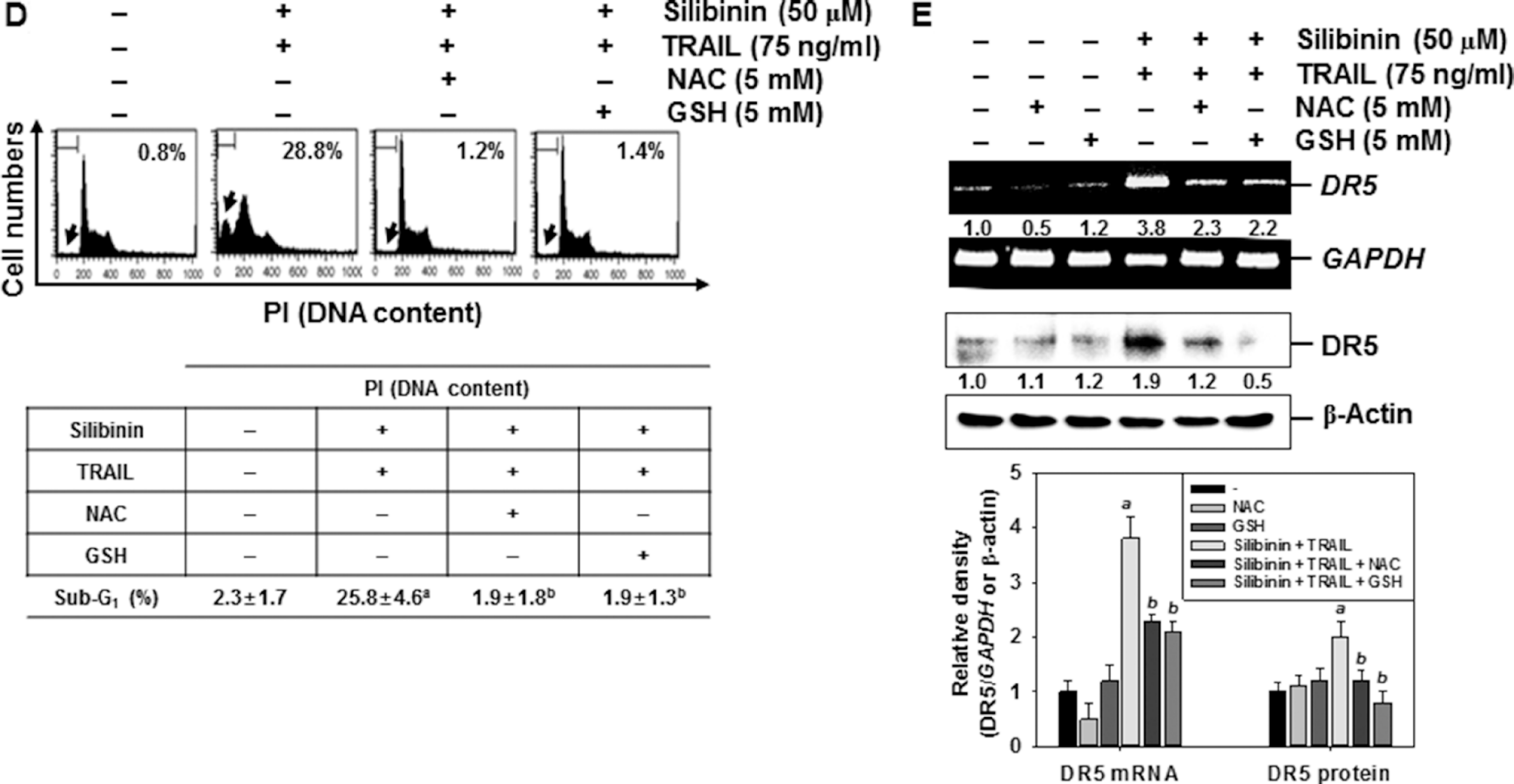
ROS-mediated DR5 expression induced by silibinin/TRAIL (**A**) A549 cells were treated with the indicated concentrations of silibinin for 24 h. H_2_DCFDA-based fluorescence intensity was measured by flow cytometry. (**B**–**E**) A549 cells were pretreated with 5 mM NAC and 5 mM GSH for 30 min and were further administrated with 50 μM silibinin and 75 ng/ml TRAIL for 24 h. ROS generation was analyzed by flow cytometry using H_2_DCFDA (B). Cell viability was measured by MTT assay (C). Sub-G_1_ cell distribution was analyzed by propidium iodide (PI) (D). Total RNA and protein were isolated, and RT-PCR analysis (top) and western blot analysis (bottom) for DR5 expression were performed (E). β-Actin and GAPDH were used as internal controls for western blot analysis and RT-PCR, respectively. Each point represents the mean ± S.E. of three independent experiments. Statistical significance was determined by a two-way ANOVA test (^*a*^*p* < 0.05 *vs*. untreated group and ^*b*^*p* < 0.05 *vs*. silibinin/TRAIL-treated group).

ROS is a key activator in DR5 upregulation, which results in TRAIL-mediated apoptosis [10, 12, 14]. Therefore, we investigated whether ROS is required for silibinin/TRAIL-induced apoptosis in A549 cells. H_2_DCFDA-based FACS analysis revealed that treatment with silibinin significantly promoted ROS generation (Figure 5A). Our results indicated that pretreating cells with the antioxidants, *N*-acetyl-L-cysteine (NAC) and glutathione (GSH), effectively blocked silibinin/TRAIL-induced ROS generation (Figure 5B).

We further evaluated the effect of silibinin/TRAIL-induced production of ROS on the viability of A549 cells. Silibinin/TRAIL decreased cell viability by approximately 55%; however, pretreatment with NAC and GSH restored cell viability by approximately 70% (Figure 5C). The results also showed that silibinin/TRAIL markedly increased the percentage of sub-G_1_ cells. However, pretreatment with NAC and GSH resulted in a significant decrease in the percentage of sub-G_1_ cells in response to silibinin/TRAIL (Figure 5D). We also determined whether ROS was involved in silibinin/TRAIL-mediated expression of DR5. Pretreatment of A549 cells with NAC and GSH markedly reduced silibinin/TRAIL-induced upregulation of DR5 at the transcription (top) and translation (bottom) levels (Figure 5E). The data obtained clearly indicate that silibinin-induced ROS generation is critical for the expression of DR5, and contributes to the sensitization of cells to TRAIL-induced apoptosis.

### Silibinin stimulates ROS-induced ER stress

Recent publications have revealed that several chemicals potentiate TRAIL-induced apoptosis in various cancer cells by stimulating ER stress, which suggests that ER stress is a good therapeutic target in TRAIL sensitization by cells [10, 12, 14]. To further understand the relationship between silibinin and ER stress, we measured the levels of ER stress and associated proteins in response to silibinin.

**Figure 6:**
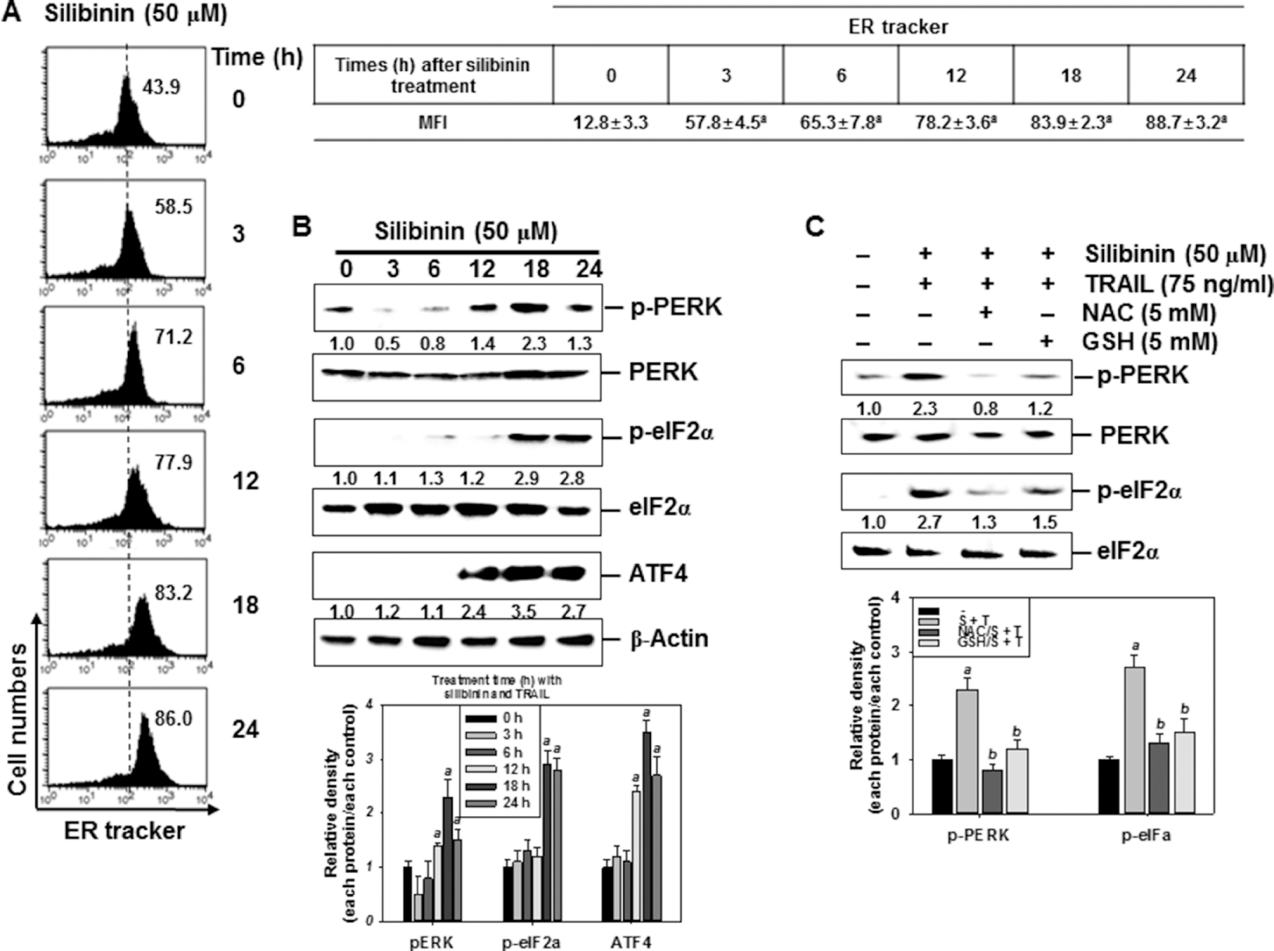
Induction of ROS-mediated ER stress in response to silibinin/TRAIL (**A–B**) A549 cells were treated with 50 μM silibinin at the indicated time-points. Fluorescence intensity of ER tracker was measured by flow cytometry (A). The cells were harvested and whole-cell protein lysates were prepared for detection of the ER stress markers by western blot analysis (B). (**C**) A549 cells were pretreated with 5 mM NAC and 5 mM GSH for 30 min and were further treated with 50 μM silibinin and 75 ng/ml TRAIL for 24 h. Total protein was isolated and western blot analysis was performed for detection of the ER stress markers. β-Actin was used as an internal control. Each point represents the mean ± S.E. of three independent experiments. Statistical significance was determined by a two-way ANOVA test (^*a*^*p* < 0.05 *vs*. untreated group and ^*b*^*p* < 0.05 *vs*. silibinin/TRAIL-treated group).

Treatment with silibinin gradually increased the intensity of ER-tracker-FITC in A549 cells in a time-dependent manner (Figure 6A). We subsequently evaluated whether the silibinin-induced ER stress was associated with the expression of ER marker proteins. Treatment with silibinin increased the phosphorylation of PERK and eIF2α, and the expression of ATF4 at 24 h, in a time-dependent manner. This confirmed that silibinin evokes ER stress in A549 cells because of the upregulation of ER marker proteins (Figure 6B). We also found that pretreatment of the cells with NAC and GSH inhibited silibinin/TRAIL-induced phosphorylation of PERK and eIF2α (Figure 6C). The obtained results imply that silibinin-induced ER stress results from the generation of ROS.

### Silibinin enhances ER stress-induced expression of CHOP by increasing ROS generation, leading to sensitivity of cells to TRAIL-induced apoptosis

**Figure 7:**
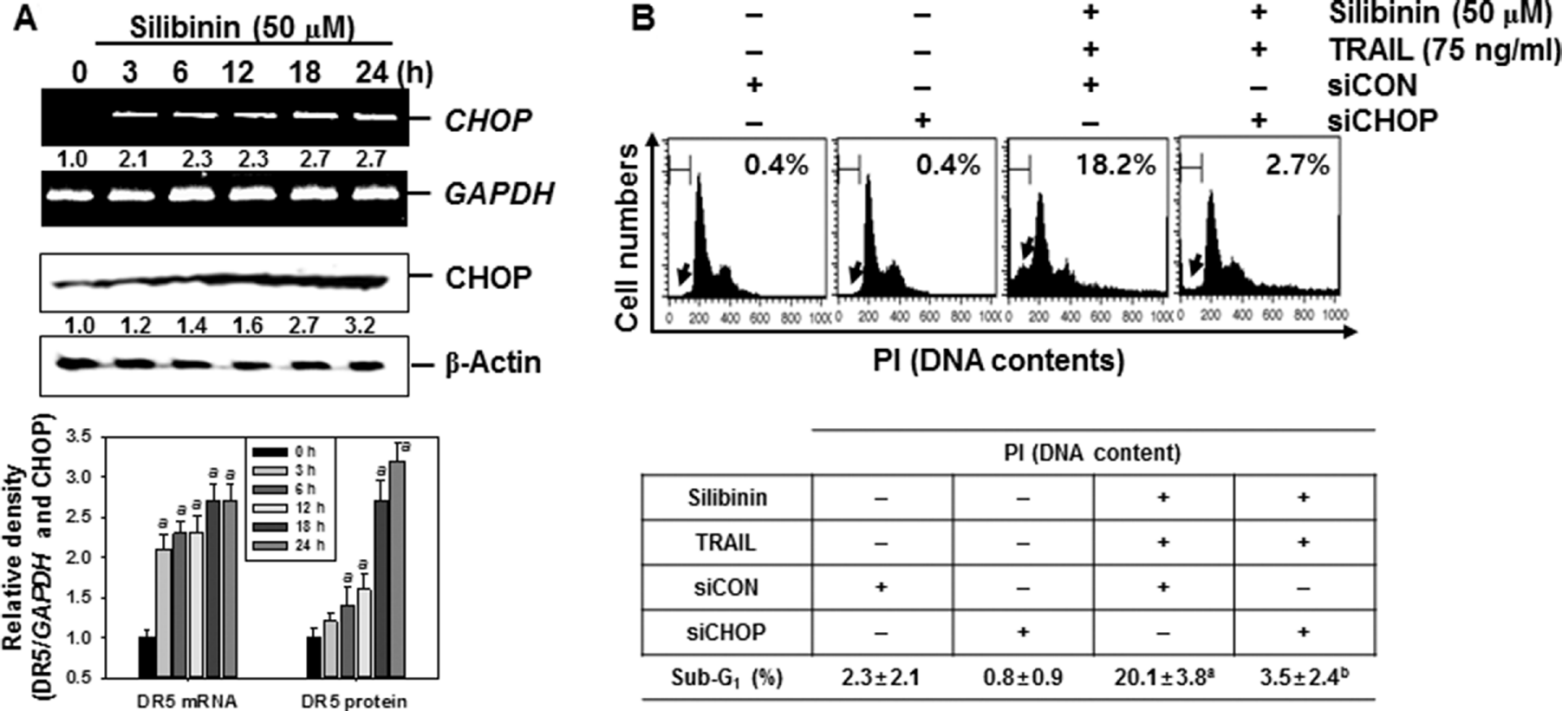

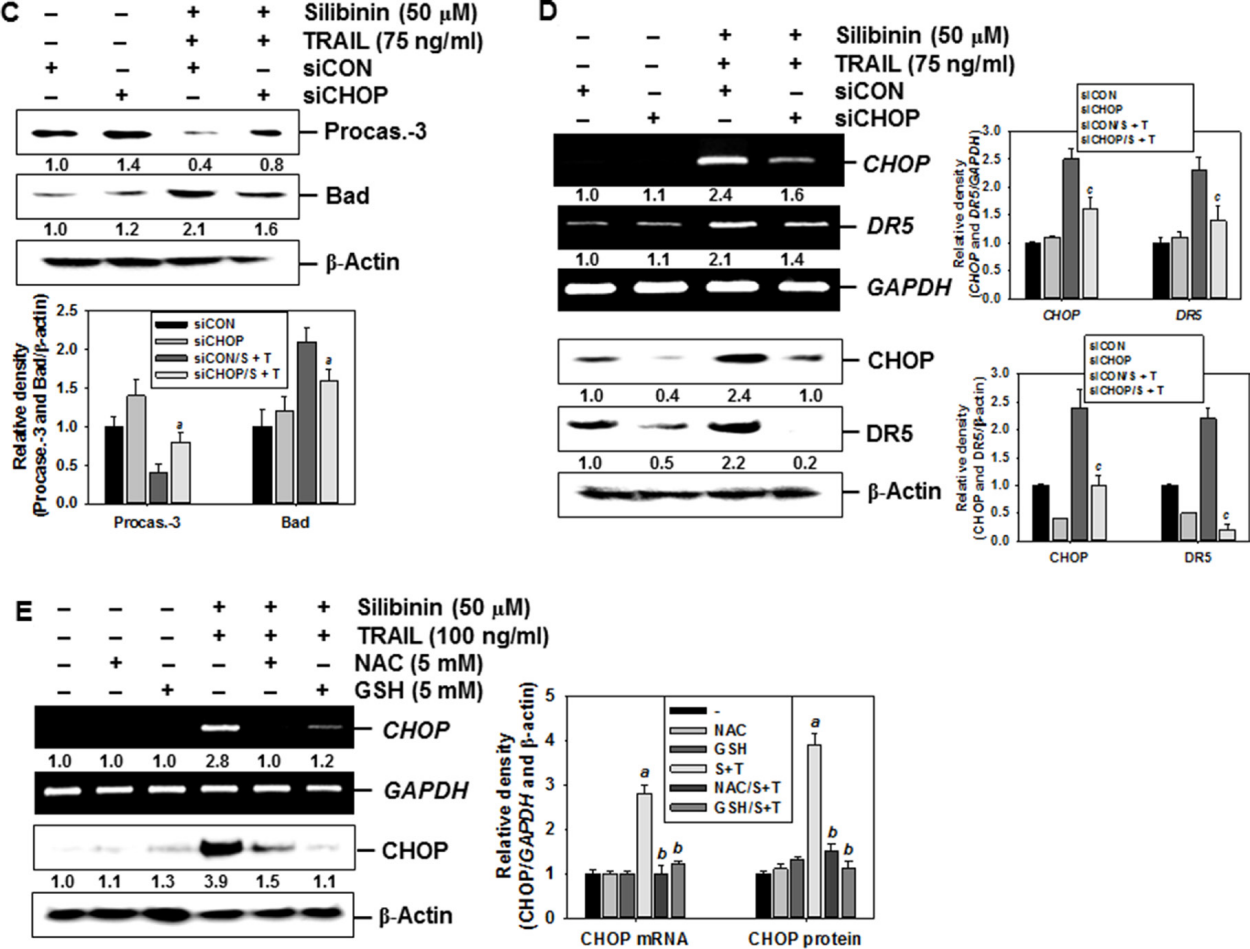
CHOP-dependent DR5 regulation by ROS-mediated ER stress in response to silibinin/TRAIL (**A**) A549 cells were treated with 50 μM silibinin at the indicated time-points. Total RNA and protein were isolated, and RT-PCR and western blot analysis for CHOP expression was performed. (**B**–**D**) A549 cells were pretreated with *CHOP* siRNA (siCHOP) for 24 h and were further treated with 50 μM silibinin and 75 ng/ml TRAIL for 24 h. Sub-G_1_ cell distribution was analyzed by flow cytometry (B). The cells were harvested and whole-cell protein lysates were prepared for detection of caspase-3 and Bad by western blot analysis (C). Total RNA and protein were isolated and RT-PCR and western blot analysis for CHOP and DR5 were performed (D). (**E**) A549 cells were pretreated with 5 mM NAC and 5 mM GSH for 30 min and then were incubated with 50 μM silibinin and 75 ng/ml TRAIL for 24 h. Total RNA and protein were isolated, and RT-PCR and western blot analysis analysis for CHOP and DR5 were conducted. GAPDH and β-Actin were used as internal controls. Each point represents the mean ± S.E. of three independent experiments. Statistical significance was determined by a two-way ANOVA test (^*a*^*p* < 0.05 *vs*. untreated group, and ^*b*^*p* < 0.05 *vs*. silibinin/TRAIL-treated group, and ^*c*^*p* < 0.05 vs. silibinin/TRAIL-treated group in cells infected with siCON).

Recent reports have demonstrated that some chemicals selectively increase TRAIL-induced apoptosis by ROS generation, resulting in CHOP-induced upregulation of DR5 [10, 12, 14]. Therefore, we analyzed the mRNA and protein expressions of CHOP in the presence of silibinin. Our data confirmed that, silibinin promoted CHOP expression at transcription (top) and translation (bottom) levels in a time-dependent manner (Figure 7A). Moreover, transient knockdown by *CHOP*-targeted siRNA (siCHOP) significantly decreased silibinin/TRAIL-induced sub-G_1_ phase (from approximately 18% to 3%) (Figure 7B). Silibinin/TRAIL decreased the expression level of procaspase-3, whereas the absence of CHOP sustained the expression of procaspsase-3 (Figure 7C). Additionally, siCHOP decreased silibinin/TRAIL-mediated proapoptotic Bad expression. These data indicate that the knockdown of CHOP significantly attenuated silibinin/TRAIL-induced apoptosis. siCHOP significantly inhibited silibinin/TRAIL-induced expression of DR5, accompanied with downregulation of CHOP expression (Figure 7D). To elucidate the relationship between ROS generation and CHOP-dependent induction of DR5 in silibinin/TRAIL-induced apoptosis, we examined whether ROS upregulates silibinin-induced CHOP in the presence of NAC and GSH. We found that pretreatment of cells with NAC and GSH significantly decreased silibinin/TRAIL-induced expression of CHOP (Figure 7E). These data suggest that silibinin-induced induction of CHOP upregulates DR5 expression, thus contributing to the sensitivity of cells to TRAIL-induced apoptosis.

### Ca^2+^ signaling pathway involves in silibinin-induced upregulation of DR5, resulting in enhancement of silibinin/TRAIL-induced apoptosis

**Figure 8:**
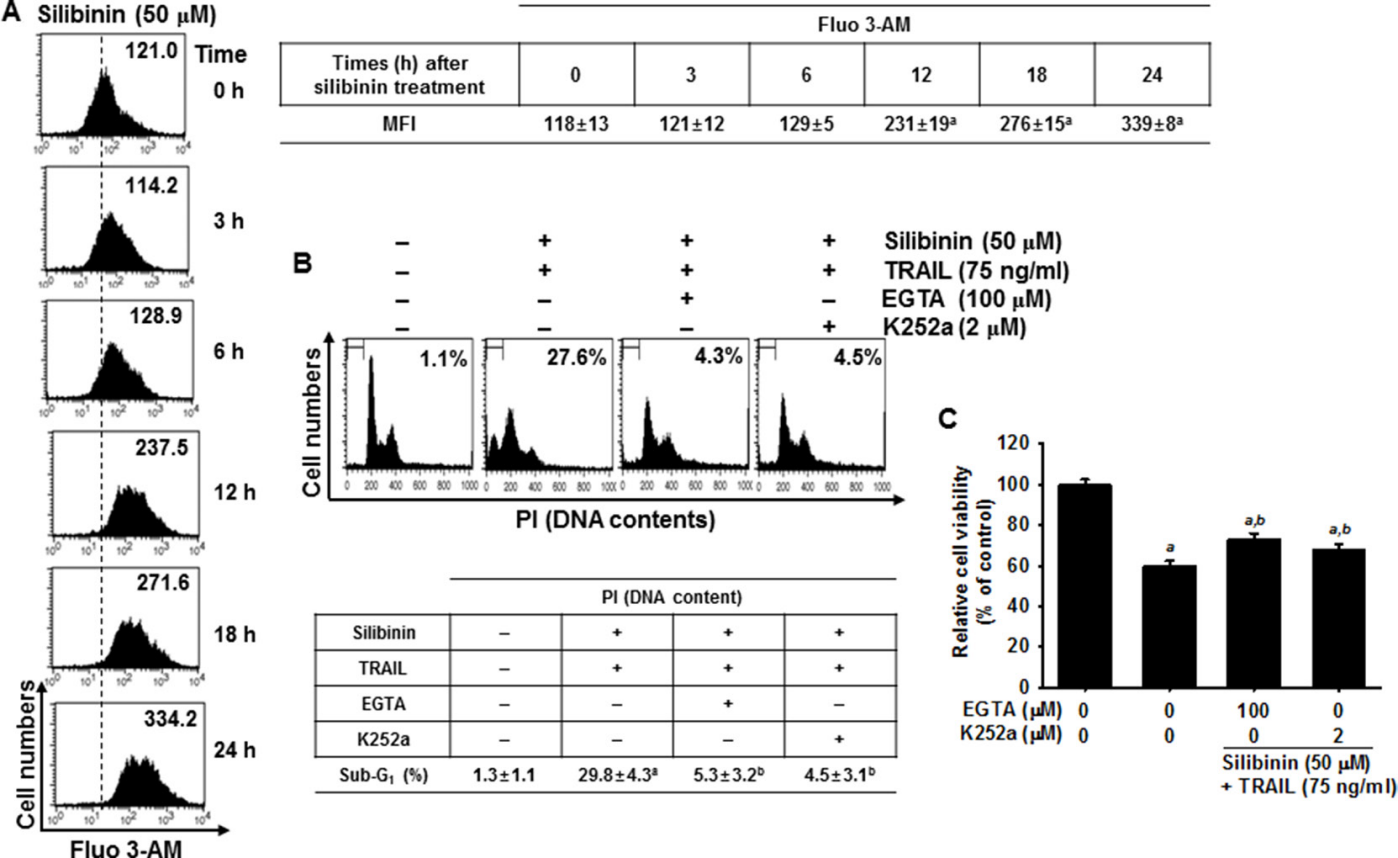

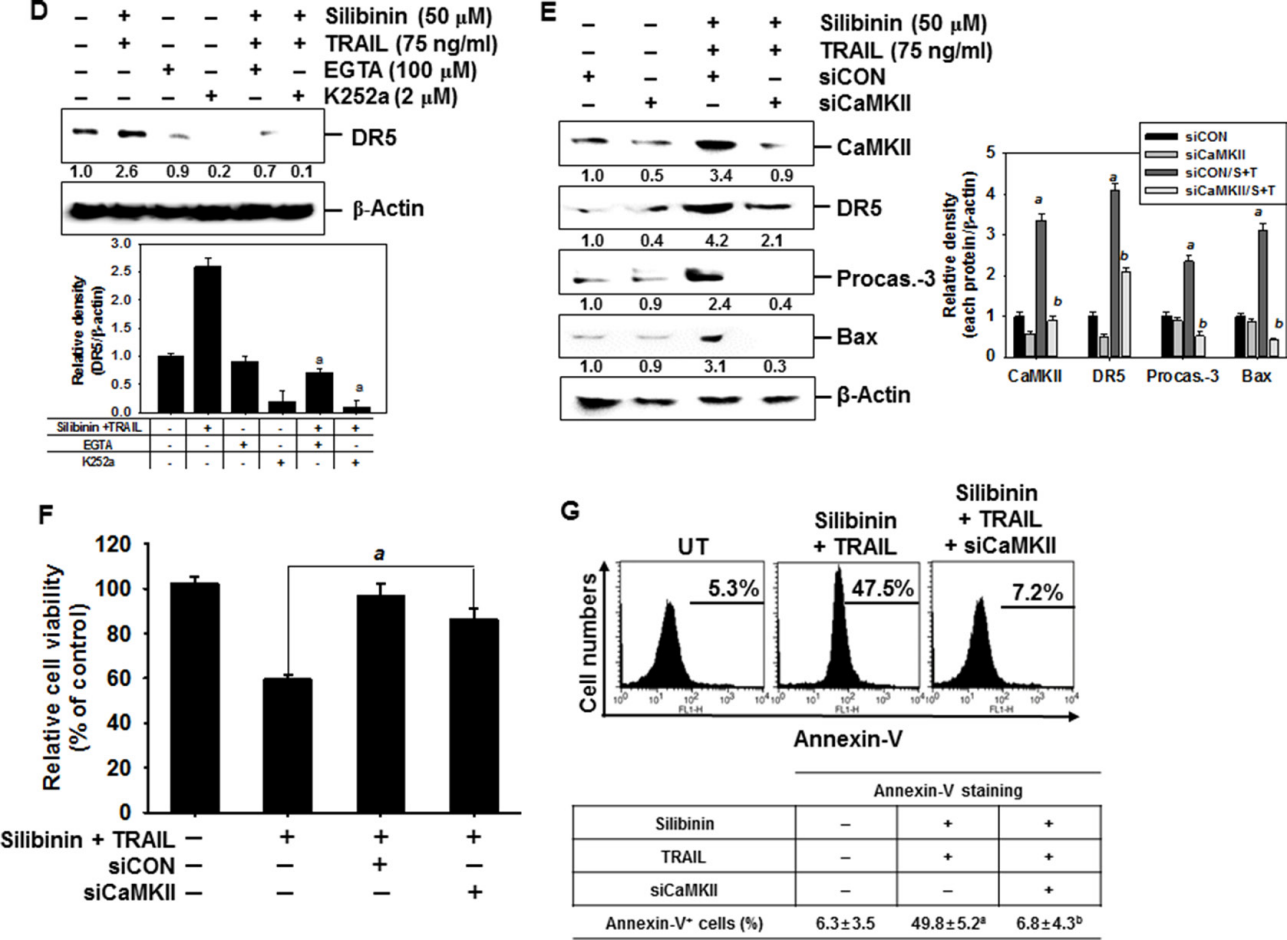
DR5 regulation by Ca^2+^-induced CaMKII in silibinin/TRAIL-mediated apoptosis (**A**) A549 cells were treated with 50 μM silibinin at the indicated time-points. Fluorescence intensity of Fluo 3-AM was measured by flow cytometry. (**B**–**D**) A549 cells were pretreated with 100 μM EGTA and 2 μM K252a for 30 min and then treated with 50 μM silibinin and 75 ng/ml TRAIL for 24 h. Sub-G_1_ cell distribution was analyzed by flow cytometry (B). Cell viability was measured by MTT assay (C). The cells were harvested and whole-cell protein lysates were prepared for detection of DR5 by western blot analysis (D). (**E**–**G**) A549 cells were pretreated with *CaMKII* siRNA (siCaMKII) for 24 h and were further treated with 50 μM silibinin and 75 ng/ml TRAIL for 24 h. Total protein was isolated and western blot analysis for CaMKII, DR5, procaspase-3, and Bax were performed (E). Cellular viability was determined by MTT assay (F). In a parallel experiment, annexin-V^+^ population of V74-9 cells (top panel) and C2C12 cells (bottom panel) was analyzed by flow cytometry (G). β-Actin was used as an internal control. Each point represents the mean ± S.E. of three independent experiments. Statistical significance was determined by two-way and one-way ANOVA test (^*a*^*p* < 0.05 *vs*. untreated group and ^*b*^*p* < 0.05 *vs*. silibinin/TRAIL-treated group).

ROS cause ER stress, which leads to activation of the downstream effector, CHOP, which then triggers Ca^2+^ release from the ER [14–16]. However, the role of silibinin/TRAIL on Ca^2+^-induced apoptotic pathways is not fully understood. To clarify the effects of silibinin on Ca^2+^ release, A549 cells were treated with silibinin and stained with Fluo 3-AM at predetermined time points. Treatment with silibinin resulted in a gradual increase in intracellular Ca^2+^ levels (Figure 8A). To explore whether silibinin enhances TRAIL-induced apoptosis through the Ca^2+^ signal pathway, we analyzed the sub-G_1_ populations and conducted MTT assays in the presence of a Ca^2+^-chelating agent, ethylene glycol tetraacetic acid (EGTA), and a Ca^2+^/calmodulin-dependent protein kinase II (CaMKII) inhibitor, K252a. The Ca^2+^ signal inhibitors, EGTA and K252a, significantly decreased silibinin/TRAIL-induced apoptotic sub-G_1_ populations (Figure 8B) and restored cell viability (Figure 8C). This indicates that the Ca^2+^ signaling pathway is involved in silibinin/TRAIL-induced apoptosis. Interestingly, the Ca^2+^ signal inhibitors also decreased silibinin/TRAIL-induced upregulation of DR5 (Figure 8D). In addition, we determined that CaMKII directly stimulates silibinin/TRAIL-induced apoptosis by upregulating DR5. Transient knockdown of CaMKII significantly decreased DR5 expression in the presence of silibinin/TRAIL accompanied with remarked downregulation of procaspase-3 and Bax (Figure 8E) and restored silibinin/TRAIL-mediated apoptosis (Figure 8F and 8G). These results confirm that silibinin enhances expression of DR5 through Ca^2+^-induced CaMKII, which is the outcome of ROS-induced ER stress.

### Silibinin enhances expression of DR5 through the activation of Sp1

**Figure 9:**
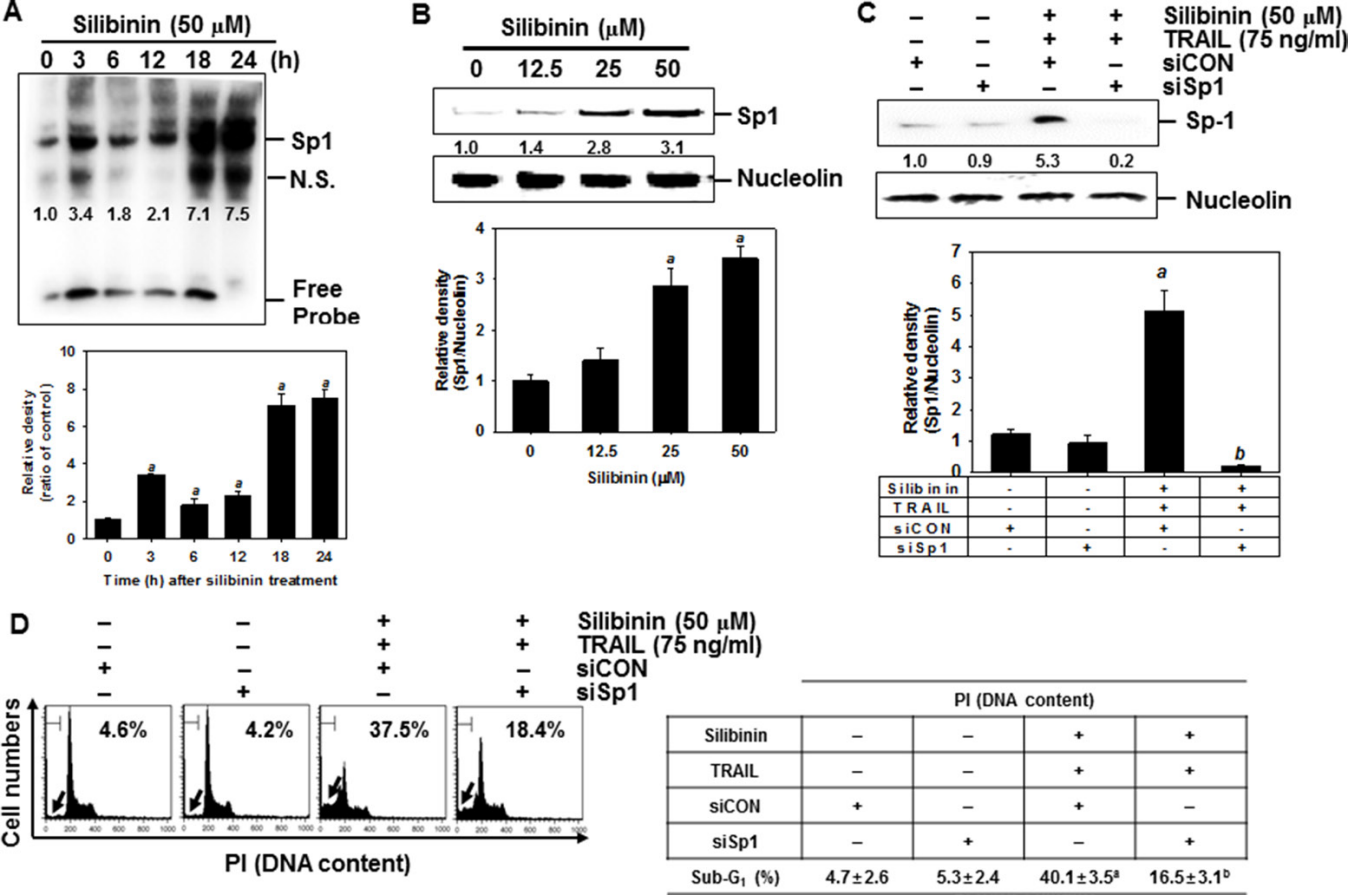

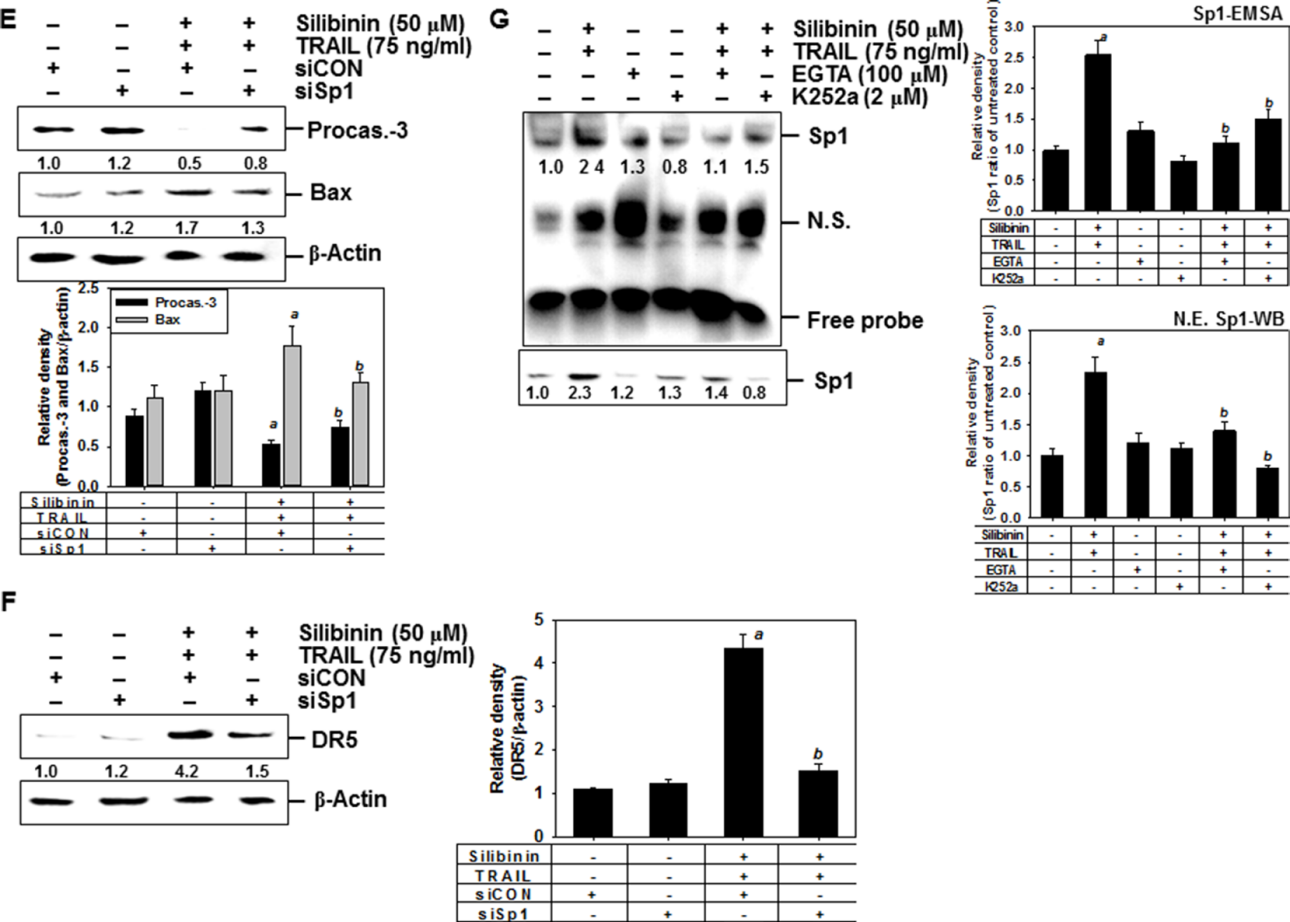
DR5 upregulation by silibinin through Sp1 activation, leading to apoptosis (**A**) A549 cells were incubated with 50 μM silibinin at the indicated time-points. DNA-binding activity of Sp1 was analyzed by lightshift^TM^ chemiluminescent EMSA kit as described in the MATERIALS AND METHODS. (**B**) A549 cells were incubated with the indicated concentrations of silibinin for 24 h. Nuclear protein was prepared and western blot analysis was conducted for Sp1. Nucleolin was used as an internal control of nuclear protein. (**C–F**) A549 cells were transfected with *Sp1* siRNA (siSp1) for 24 h and then the cells were treated with silibinin and/or TRAIL for 24 h. Nuclear protein was prepared and western blot analysis was conducted for Sp1 (C). Sub-G_1_ cell distribution was analyzed by flow cytometry (D). Cell extracts were prepared for western blot analysis for caspase-3 and Bad (E). Western blot analysis was also performed for DR5 expression (**G**). β-Actin was used as an internal control. G. A549 cells were pretreated with 100 μM EGTA and 2 μM K252a for 2 h and then treated with 50 μM silibinin and 75 ng/ml TRAIL for 24 h. The nuclear extracts were assayed for DNA-binding activity of Sp1 by electrophoretic mobility shift assay (EMSA) (top). The nuclear protein was prepared and western blot analysis was performed for Sp1 (bottom). Each point represents the mean ± S.E. of three independent experiments. Statistical significance was determined by two-way and one-way ANOVA test (^*a*^*p* < 0.05 *vs*. untreated group and ^*b*^*p* < 0.05 *vs*. silibinin/TRAIL-treated group).

Yoshida *et al*. reported that the DR5 promoter region containing spanning nucleotides −605 to +3 for transcription factor binding sites to Sp1, which enhances TRAIL-mediated apoptosis by overexpression of DR5 [21]. Therefore, to investigate the role of silibinin on the expression of Sp1, EMSA assay was performed to ascertain the DNA binding activity of Sp1 in A549 cells. The results showed that silibinin treatment promoted the DNA binding activity of Sp1 in a time-dependent manner (Figure 9A). We further investigated whether silibinin upregulates the nuclear translocation of Sp1 by western blot analysis in A549 cells. Silibinin increased nuclear levels of Sp1 in a dose-dependent manner (Figure 9B). The functional role of Sp1 in silibinin/TRAIL-mediated apoptosis was then evaluated by performing a transient knockdown of Sp1 in A549 cells, using siSp1. As presumed, siSp1 significantly downregulated nuclear Sp1 (Figure 9C). In addition, sub-G_1_ analysis showed that silibinin/TRAIL-mediated apoptosis was enhanced by 37.5%; however, the presence of siSp1 prevented cytotoxicity by 18.4% in the A549 cells (Figure 9D). Additionally, the western blot data showed that the transient knockdown of Sp1 restored silibinin/TRAIL-mediated downregulation of procaspase-3, and decreased apoptotic Bad expression (Figure 9E). This means that Sp1 is an important transcription factor in silibinin/TRAIL-induced apoptosis.

We also investigated whether silibinin stimulated DR5 expression via upregulation of Sp1. Transient knockdown of Sp1 in A549 cells resulted in loss of protein expression of DR5, induced by silibinin/TRAIL (Figure 9F). Because CaMKII showed a direct modulation of Sp1 transcriptional activity [22, 23], we hypothesized that silibinin-induced Sp1 activity may be due to the activation of CaMKII. To evaluate this possibility, we pretreated cells with EGTA and K252a, and examined the DNA binding activity of Sp1 using EMSA assay. The inhibitors effectively blocked the DNA binding activity of Sp1 in response to silibinin/TRAIL (Figure 9G, top). Similarly, western blot analysis also confirmed that silibinin/TRAIL-induced nuclear Sp1 was inhibited in the presence of EGTA and K252a (Figure 9G, *bottom*). These results confirm that silibinin activates DR5 expression through CaMKII-induced Sp1 expression, which increases cell sensitivity to TRAIL

## DISCUSSION

**Figure 10:**
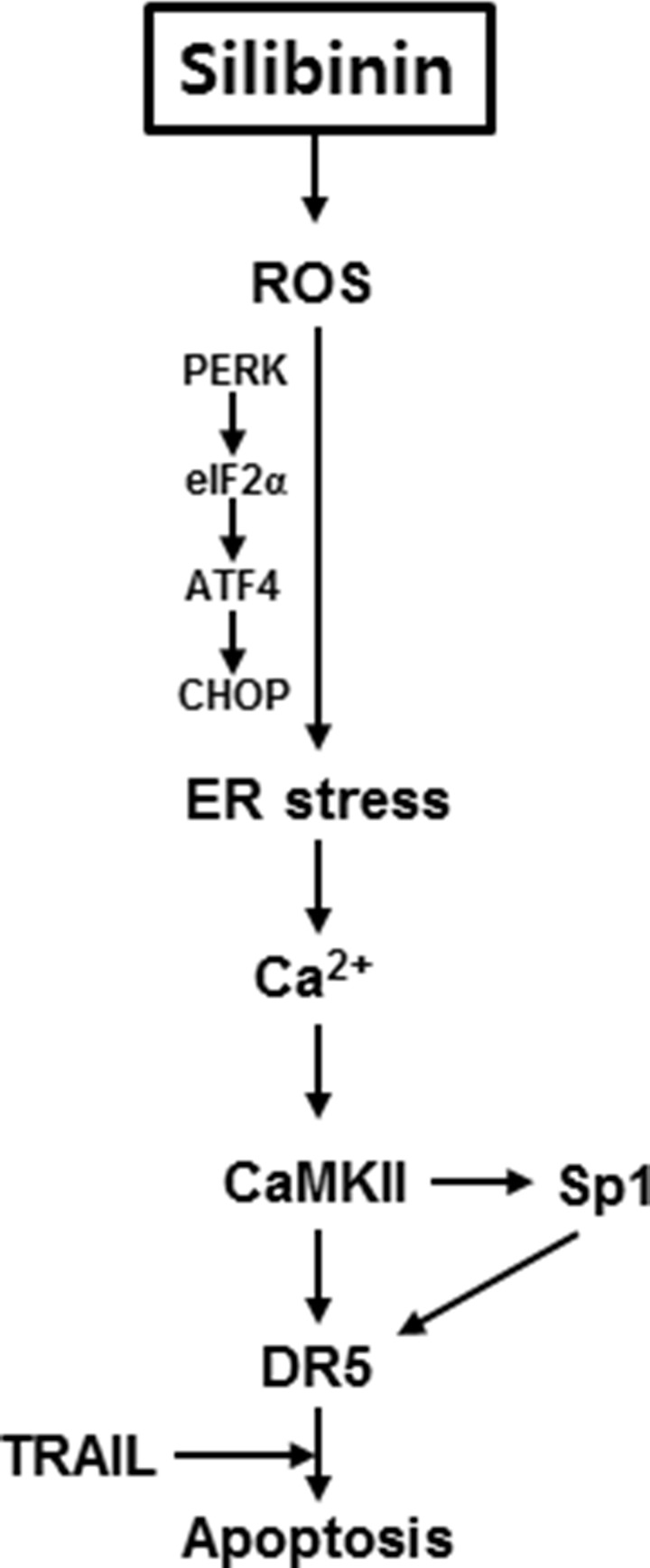
Schematic explanation of the multiple signal pathways involved in controlling DR5 expression in response to silibinin/TRAIL Binding of TRAIL to DR5 leads to activation of the caspase cascade system, and silibinin enhances TRAIL sensitivity for apoptosis. Silibinin stimulates ER stress along with PERK-eIF2α-ATF4, which may open Ca^2+^ channels on the ER by activating CHOP. Consequently, cytosolic Ca^2+^ influx in response to silibinin induces Ca^2+^-sensitive CaMKII. CaMKII directly upregulates DR5 expression or indirectly links with the Sp1 pathway, causing sensitization of cells to TRAIL-mediated apoptosis.

TRAIL is regarded as a potential anticancer agent [2]. Many cancer cells were originally sensitive to TRAIL-mediated apoptosis whereas some cancer cells, especially highly malignant tumors were strongly resistant to TRAIL [24]. Therefore, combined treatments with many natural products, histone deacetylase inhibitors, and proteasome inhibitors have been identified to sensitize cancer cells to TRAIL-mediated apoptosis [25, 26]. Recent reports have shown that upregulation of DR5 is a promising therapeutic strategy to prevent the resistance of cancer cells to TRAIL and result in TRAIL-resistant cancer cells [27]. In previous, many publications showed that silibinin stimulated TRAIL-induced apoptosis by inducing DR5 expression *in vivo* and *in vitro*, which blocks activation of c-FLIP and survivin, and stimulates activation of caspases via mitochondrial or non-mitochondrial pathways [28, 29, 30]. In the present study, treatment with silibinin also resulted in a time-dependent increase in DR5 expression and DR5/Fc chimera protein effectively inhibited silibinin/TRAIL-induced apoptosis, suggesting that silibinin is a promising sensitizer of TRAIL-mediated apoptosis by activating DR5 expression. Nevertheless, no research has been reported so far whether combination treatment with silibinin and TRAIL stimulates ER stress and ER stress-downstream molecules are related to silibinin/TRAIL-induced apoptosis (Figure 10). Therefore, we, in the current study, determined to track ER stress and its downstream molecules in silibinin/TRAIL-induced apoptosis.

Accumulation of intracellular ROS leads to disruption of the mitochondrial membrane potential, increases the release of cytochrome *c* into the cytosol, and subsequently activates caspases for apoptosis [31]. Recent studies have also found that some chemopreventive agents trigger and amplify TRAIL-induced apoptosis by upregulating DR5 expression via ROS generation [32, 33]. Kim *et al*. reported that silibinin caused an increase in ROS generation in malignant cells and resulted in caspase-3-induced apoptosis [19]. Nevertheless, how silibinin-induced generation of ROS sensitizes cells to TRAIL-mediated apoptosis is not understood. Therefore, in the present study, we provided a novel mechanism by which silibinin-induced upregulation of DR5 occurs for the sensitization of cells to TRAIL-mediated apoptosis.

Based on our data, silibinin also stimulated TRAIL-mediated apoptosis by ROS-induced DR5 expression and NAC and GSH significantly prevented silibinin/TRAIL-induced apoptosis, accompanied with a low expression of DR5. This suggests that ROS is a key activator in silibinin/TRAIL-induced apoptosis. Kauntz *et al.* previously determined the cytotoxic effect of silibinin in colon adenocarcinoma cells and its anti-metastatic activity *in vivo* and *in vitro* without any adverse effects, resulting from enhancement of ROS generation [29, 30]. Therefore, previous and our current data could support that silibinin is a promising candidate of TRAIL sensitizer, although combination with silibinin and TRAIL has not been evaluated *in vivo*. Recently, Sinha *et al*. highlighted that ROS production and their sites of formation are directly involved in the survival or death of various cancer cells and help to find strategic therapeutics in future [34]. In particular, Circu *et al*. showed how redox system controls apoptotic initiation and execution on death receptor-mediated and mitochondrial pathways of cell death by promoting ROS generation [35]; extrinsic stimuli such as TRAIL promotes cytoplasmic ROS generation, which engages mitochondrial apoptotic signaling and oxidants such as silibinin directly permeates into mitochondrial outer membrane and sensitizes ROS generation. Many researches confirmed that silibinin itself enhanced ROS generation from mitochondria, which can cause apoptosis via oxidative stress [36, 37]. In the current study, we also found that silibinin itself increased ROS generation and silibinin/TRAIL significantly enhanced the generation than that of treatment with silibinin itself, which indicates that combined treatment with silibinin and TRAIL could robustly upregulated ROS generation from cytoplasm by TRAIL and mitochondria by silibinin, causing to multiply apoptotic signal. Previous many publications showed whether chemicals and drugs stimulate ROS generation and DR5 expression in response to TRAIL; however, we, furthermore, focused on which downstream molecules activate the expression of DR5 in silibinin/TRAIL-mediated generation of ROS.

ER stress leads to aberrant homeostasis in the ER [38], resulting in unexpected neurodegenerative disorders, tumorigenesis, and metabolic diseases. These occur by the accumulation of unfolded protein responses in the ER lumen. If ER stress is not resolved, apoptosis is induced [39]. Three specific response marker proteins such as PERK, ATF6, and inositol-requiring enzyme 1 (IRE1) stimulate ER stress. The PERK signal pathway is particularly well-known to induce translational attenuation by activating the eIF2α-ATF4-CHOP axis [40]. In the present study, we found that silibinin markedly triggered ER stress via generation of ROS. This was because NAC and GSH significantly inhibited the expression of silibinin-induced ER marker proteins such as PERK, ATF4, and eIF2α. Additionally, excessive ER stress promotes Ca^2+^ leakage from the ER lumen, causing to unfolded protein response through CHOP upregulation. An alteration of mitochondrial membrane potential also occurs, which potently stimulates the expression of apoptotic proteins such as Bad and caspase-3 [41]. From our previous studies, Ca^2+^ depletion is required in ER-induced apoptosis in a CHOP-dependent manner, by stimulating mitochondrial membrane potential. We also showed that siCHOP inhibited silibinin/TRAIL-mediated apoptosis. This event was accompanied with downregulation of caspase-3 and Bad, via suppression of DR5 expression. This suggests that silibinin sensitizes TRAIL-mediated apoptosis by activating CHOP-induced upregulation of DR5. Some previous studies showed that silibinin promoted ROS generation as a pro-oxidant, and led to apoptosis via multiple pathways. This suggests that silibinin is a promising candidate for the treatment of a number of cancers [14–20]. However, the antioxidant effect of silibinin was also shown to attenuate pulmonary injury [41]. The aforementioned studies suggest that silibinin possesses a dual-faced effect as a pro-oxidant and an antioxidant, depending on the type of cancer or diseases for which silibinin is used. Therefore, the molecular mechanisms and effects of silibinin should be investigated in different cancer cells and animal models. In the present study, we first demonstrated that ROS triggered ER stress in response to silibinin, along the PERK-eIF2α-ATF4-CHOP pathway. This then led to TRAIL-mediated apoptosis via upregulation of DR5.

As previously mentioned, from ER due to silibinin/TRAIL disturbs mitochondrial membrane potential, which is responsible for the expression of apoptotic proteins. The Ca^2+^ leakage is accompanied with the generation of ROS [17–20]. However, there is no evidence as to whether the Ca^2+^ signaling pathway preliminarily enhances ROS generation. On the other hand, many recent studies have supported excessive generation of ROS via cellular oxidation, and cellular metabolism is required for Ca^2+^ dysregulation [42, 43]. In the present study, we determined that silibinin/TRAIL promotes ROS generation and causes ER stress, which stimulates the Ca^2+^ signaling pathway. Ca^2+^-sensitive CaMKII is a main downstream molecule in the Ca^2+^ signal pathway, which leads to apoptosis in numerous cancer cells. CaMKII is therefore a promising target for the diagnosis and treatment of cancer [44]. Additionally, Ca^2+^ signaling via ROS generation has been thought as a novel therapeutic target for preventing inflammatory diseases [39, 40]. In the current study, we found that silibinin/TRAIL activated Ca^2+^-induced CaMKII is also linked to ER stress. The activation was enhanced by generation of ROS for the mitochondrial apoptotic pathways through DR5 upregulation. Sohm *et al*. previously reported that the Ca^2+^-induced CaMKII pathway stimulates cell cycle distribution and apoptosis through the Sp1 signal pathway [45, 46]. Similarly, previous experiments demonstrated that TRAIL-mediated Sp1 activation increases the sensitivity of cancer cells to apoptosis through the upregulation of DR5 expression [16, 47]. Thus, Sp1 activation is an important target in TRAIL-resistant cancer cells to strengthen its sensitivity through upregulation of DR5 by binding to the transcription start sites of DR5. This suggests that the ROS-ER stress-Ca^2+^-CaMKII axis is critical for the expression of DR5 [8]. Previously, we have reported that the mutation of the Sp1-binding site on the DR5 promoter markedly inhibited the transcriptional activation of DR5. In addition, reporter gene analysis showed that two regions, −305 and −300, of the DR5 promoter are important in DR5 promoter activity [9]. However, whether CaMKII directly activates Sp1 activity in the expression of DR5 is yet to be evaluated. We found that treatment with silibinin significantly induced Sp1 activation, through the ROS-induced ER stress-Ca^2+^-CaMKII axis. However, we needed to elucidate which region of the DR5 promoter was important for silibinin-induced Sp1 upregulation. This is because Sp1 can also bind to the −195 and −159 regions of DR5 promoter. Although Sp1 is the main regulator of DR5 transcription, it is the possible that other transcription factors including NF-κB, p53, and Yin Yang 1 bind to the −305 and −300 regions of the DR5 promoter region, [48, 49].

In conclusion, the present study provided the first mechanistic evidence that silibinin effectively restores TRAIL sensitivity of cells, through ROS-induced ER stress. Furthermore, silibinin/TRAIL-induced ROS generation linked ER stress with CHOP-induced DR5 upregulation (Figure 10). Additionally, silibinin potentiates the apoptotic effects of TRAIL via the Ca^2+^-CaMKII pathway, which also stimulates Sp1 activity. From our results, combined treatment with silibinin and TRAIL may be a novel strategy for the treatment of a variety of human cancers that are resistant to chemotherapy.

## MATERIALS AND METHODS

### Regents and antibodies

Silibinin, 3-(4,5-dimethylthiazol-2-yl)-2,5-diphenyltetrazolium bromide (MTT), propidium iodide (PI), glutathione (GSH), *N*-acetyl-L-cysteine (NAC), ethylene glycol tetraacetic acid (EGTA), and K252a were purchased from Sigma (St. Louis, MO) and an enhanced chemiluminescence (ECL) kit was purchased from Amersham (Arlington Heights, IL, USA). 6-Carboxy-2′,7′-dichlorofluorescein diacetate (H_2_DCFDA), dihexyloxacarbocyanine iodide (DiOC_6_), ER tracker, and Fluo 3-AM were purchased from Molecular Probes (Eugene, OR, USA). PD98059, SP600125, SB239063, z-VAD-fmk, and z-IETD-fmk were purchased from Calbiochem (San Diego, CA, USA). RPMI1640 medium, fetal bovine serum (FBS), and antibiotics mixture were purchased from WelGENE (Daegu, Republic of Korea). Antibodies against caspase-3, caspase-8, caspase-9, β-actin, nucleolin, DR5, Sp1, IAP-1, IAP-2, Bcl-2, Bax, phospho (p)-PERK, PERK, p-eIF2α, eIF2α, ATF4, and CHOP were purchased from Santa Cruz Biotechnology (Santa Cruz, CA). Peroxidase-labeled donkey anti-rabbit and sheep anti-mouse immunoglobulin, and recombinant human TRAIL/Apo2 ligand (the nontagged 19-kDa protein, amino acids 114–281) purchased from KOMA Biotechnology. DR5/Fc chimera protein was purchased from R&D Systems (Minneapolis, MN).

### Cell culture and viability assay

Human lung cancer (A549 and NCI-H460) cells, human leukemia U937 cells, hamster fibroblast V79-4 cells, and mouse myoblast C2C12 cells were maintained in RPMI1640 supplemented with 10% heat-inactivated FBS and 1% antibiotics mixture. For analysis of cell viability, the cells were seeded at the density of 1 × 10^5^ cells/ml and then treated with the indicated concentrations of silibinin and TRAIL in the presence or presence of z-VAD-fmk, anti-DR5, PD98059, SP600125, SB239063, NAC, GSH, EGTA, or K252a for 24 h. MTT was subsequently added to each well. After 1 h-incubation, formazan was dissolved with DMSO and the absorption at 540 nm was determined by an ELISA plate reader.

### Flow cytometry analysis

A549 cells were plated at a density of 1 × 10^5^ cells/well for overnight and treated with 75 ng/ml TRAIL either in the absence or presence of silibinin for 24 h. Apoptotic cells were determined by the annexin-V^+^ staining (R&D Systems). In brief, the cells were stained with annexin-V, incubated for 15 min at room temperature in the dark, and immediately analyzed according to the manufacturer′s instructions. For analysis of apoptotic sub-G_1_ fraction, the cells were fixed by RNase A (DNase free) and PI overnight in the dark at room temperature. A FACSCalibur flow cytometer (Becton Dickinson, San Jose, CA, USA) was used to determine the number of apoptotic cells, i.e., cells with sub-G_1_ DNA that were annexin-V^+^.

### Analysis of cell morphology

A549 cells were plated at a density of 1 × 10^5^ cells/well for overnight and treated with TRAIL (75 ng/ml) and silibinin (50 μM) in the absence or presence of z-IETD-fmk. After 24 h-incubation, cell morphology was observed under light microscopy.

### Western blot analysis

Whole-cell lysates were prepared by PRO-PREP protein extraction solution (iNtRON Biotechnology, Sungnam, Republic of Korea). Cytoplasmic and nuclear protein extracts were prepared using NE-PER nuclear and cytosolic extraction reagents (Pierce, Rockford, IL, USA). The cell lysates were harvested from the supernatant after centrifugation at 13,000 *g* for 20 min. Total cell proteins were separated on polyacrylamide gels and standard procedures were used the transfer them the nitrocellulose membranes. The membranes were developed using an ECL reagent.

### Determination of mitochondrial membrane potential

The mitochondrial membrane potential was monitored by measuring the uptake of DiOC_6_. Briefly, the cells were harvested, loaded with 50 nM DiOC_6_ at 37°C for 30 min in the dark, and then analyzed using a flow cytometer.

### Luciferase assays

A549 cells were seeded at a density of 2 × 10^5^ cells/ml and grown overnight. The cells were cotransfected with pDR5/Sac I plasmid containing a DR5 promoter sequence (−250/+3) and pCMV-*β*-galactosidase plasmid using the FuGENE 6 Transfection Reagent (Roche Molecular Biochemicals, Indianapolis, IN, USA) according to the manufacturer′s instructions. After transfection, the cells were cultured in 10% FCS medium with a vehicle or drugs for 24 h. Luciferase and β-galactosidase activities were assayed according to the manufacturer's protocol (Promega). Luciferase activity was normalized with β-galactosidase activity in the cell lysate and expressed as an average of the results of 3 independent experiments.

### RT-PCR analysis

Total RNA was extracted using the TRIzol reagent (Invitrogen, Carlsbad, CA, USA) and RT-PCR was conducted following the manufacture′s protocol. cDNA was synthesized from 2 μg total RNA using MMLV reverse transcriptase. The sequences of the sense and antisense primers for *DR5* were 5′-GTC TGC TCT GAT CAC CCA AC-3′ and 5′-CTG CAA CTG TGA CTC CTA TG-3′ respectively. *CHOP* sense 5′-CAA CTG CAG AGA TGG CAG CTG A-3′, *CHOP* antisense 5′-GTC TTC ACC ACC ATG GAG-3′, *glyceraldehydes-3-phosphate dehydrogenase* (*GAPDH*) sense 5′-GTC TTC ACC ACC ATG GAG-3′, and *GAPDH* antisense 5′-CCA CCC TGT TGC TGT AGC-3′. The reaction sequence consisted of 52°C for 30 min, 94°C for 2 min, and 94°C for 35 cycles of 15s each; 61°C for 30s; and 72°C for 45 s with an extension at 72°C for 10 min. PCR products were analyzed by agarose gel electrophoresis and visualized by ethidium bromide.

### Analysis of cell surface DR5 expression

A549 cells were stained with FITC-conjugated rabbit monoclonal anti-human DR5 for 45 min at 4°C according to the manufactures protocol and analyzed by flow cytometry with FITC-conjugated IgG.

### Immunofluorescence staining

A549 cells were seeded on glass coverslips and incubated for 24 h at 37°C with or without silibinin, washed twice with PBS, and fixed with 90% methanol at 37°C for 30 min. The cells were again washed with PBS, blocked in 10% normal goat serum for 1 h, and incubated with DR5 overnight at 4°C. Primary antibody was removed by washing the membranes in PBS containing Triton-X (0.3%) and incubated for 1 h with Alexa 488-conjugated anti-mouse secondary antibody (1:200). Fluorescent signals were imaged using a confocal laser scanning microscope.

### Electrophoretic mobility shift assay

DNA-protein binding assays were carried out with a nuclear extract. Synthetic complementary Sp1-binding oligonucleotides (5′-ATT CGA TCG GGG CGG GGC GAG C-3′) were 3′-biotinylatedusing the biotin 3′-end DNA labeling kit (Pierce) according to the manufacturer′s instructions. Samples were loaded onto native 4% polyacrylamide gels pre-electrophoresed for 60 min in 0.5X Tris borate/EDTA (TBE) buffer on ice, in the presence of transferred onto a positively charged nylon membrane (Hybond^TM^-N+) in 0.5X TBE buffer at 100 V for 1 h on ice. The transferred DNA-protein complex was cross-linked to the membrane at 120 mJ/cm^2^. Horseradish peroxidase-conjugated streptavidin was utilized according to the manufacturer′s instructions to monitor the transferred DNA-protein complex.

### Small interfering RNA (siRNA)

A549 cells were seeded on a 24-well plate at a density of 1 × 10^5^ cells/ml and transfected *Sp1*-, *p38*-, *CHOP*- and *CaMKII*-specific silencing RNA (siRNA, Santa Cruz Biotechnology) for 24 h. For each transfection, 450 μl of growth medium was added to 20 nM siRNA duplex with the transfection reagent G-Fectin (Genolution Pharmaceuticals Inc., Seoul, Republic of Korea) and the entire mixture was added gently to the cells.

### Determination of ROS

Time course experiment was performed to compare ROS production in A549 cells, after treatment with various concentrations of silibinin for 24 h. A549 cells were seeded on 24-well plate at a density of 1 × 10^5^ cells/ml, preincubated with cell-permeable H_2_DCFDA florescence dye for 1 h, and then treated each compound. DCFDA fluorescence intensity was also analyzed by a FACSCalibur flow cytometry.

### ER tracker assay

A549 cells were seeded at a density of 1 × 10^5^ cells/ml and grown overnight. For ER staining, FITC-conjugated ER-Tracker was diluted 1:1000 in the regular medium. Then, the pre-warmed probe-containing medium was added to the cells and incubated for 30 min. After the cells were washed with PBS, fluorescence microscope was used to analyze.

### Ca^2+^ influx assay

The intracellular free Ca^2+^ was measured with Fluo 3-AM dye. After A549 cells were stained with Fluo 3-AM for 30 min at room temperature, the cells were treated with the indicated concentration of silibinin and fluorescence intensity was obtained using flow cytometry.

### Statistical analysis

The images were visualized with Chemi-Smart 2000 (VilberLourmat, Marine, Cedex, France). Images were captured using Chemi-Capt (VilberLourmat) and transported into Photoshop. All bands were shown a representative obtained in three independent experiments and quantified by Scion Imaging software (http://www.scioncorp.com). Statistical analyses were conducted using SigmaPlot software (version 12.0). Values were presented as mean ± standard error (S.E.). Significant differences between the groups were determined using the unpaired one-way and two-way ANOVA with Bonferroni pos*t*-test. Statistical significance was regarded at ^a^ and ^b^, *p* < 0.05.
